# Two alternative pathways for docosahexaenoic acid (DHA, 22:6n-3) biosynthesis are widespread among teleost fish

**DOI:** 10.1038/s41598-017-04288-2

**Published:** 2017-06-20

**Authors:** Angela Oboh, Naoki Kabeya, Greta Carmona-Antoñanzas, L. Filipe C. Castro, James R. Dick, Douglas R. Tocher, Oscar Monroig

**Affiliations:** 1 Institute of Aquaculture, Faculty of Natural Sciences, University of Stirling, Stirling, FK9 4LA Scotland UK; 2 Department of Biological Sciences, University of Abuja, P.M.B. 117 Abuja, Nigeria; 3 Department of Aquatic Bioscience, The University of Tokyo, 1-1-1 Yayoi, Bunkyo-ku Tokyo, 113-8657 Japan; 4 CIIMAR – Interdisciplinary Centre of Marine and Environmental Research, U. Porto – University of Porto, Porto, Portugal; 5 Department of Biology, Faculty of Sciences, U. Porto - University of Porto, Porto, Portugal

## Abstract

Docosahexaenoic acid (DHA) plays important physiological roles in vertebrates. Studies in rats and rainbow trout confirmed that DHA biosynthesis proceeds through the so-called “Sprecher pathway”, a biosynthetic process requiring a Δ6 desaturation of 24:5n−3 to 24:6n−3. Alternatively, some teleosts possess fatty acyl desaturases 2 (Fads2) that enable them to biosynthesis DHA through a more direct route termed the “Δ4 pathway”. In order to elucidate the prevalence of both pathways among teleosts, we investigated the Δ6 ability towards C_24_ substrates of Fads2 from fish with different evolutionary and ecological backgrounds. Subsequently, we retrieved public databases to identify Fads2 containing the YXXN domain responsible for the Δ4 desaturase function, and consequently enabling these species to operate the Δ4 pathway. We demonstrated that, with the exception of Δ4 desaturases, fish Fads2 have the ability to operate as Δ6 desaturases towards C_24_ PUFA enabling them to synthesise DHA through the Sprecher pathway. Nevertheless, the Δ4 pathway represents an alternative route in some teleosts and we identified the presence of putative Δ4 Fads2 in a further 11 species and confirmed the function as Δ4 desaturases of Fads2 from medaka and Nile tilapia. Our results demonstrated that two alternative pathways for DHA biosynthesis exist in teleosts.

## Introduction

**Figure 1:**
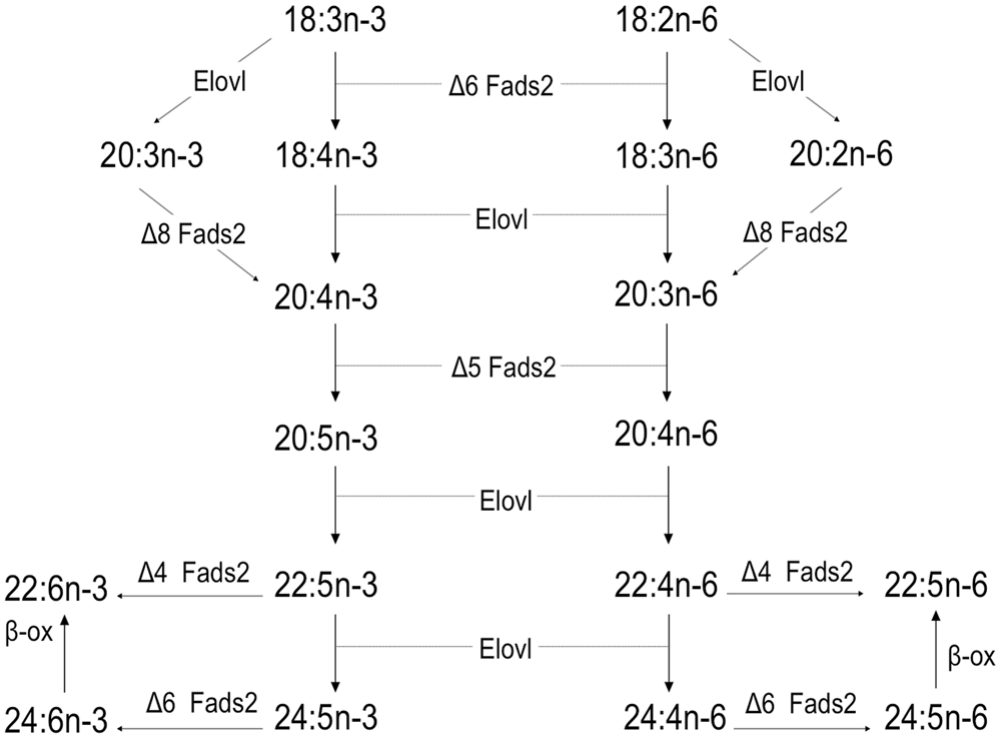
The biosynthetic pathways of long-chain (≥C_20_) polyunsaturated fatty acids from α-linolenic (18:3n−3) and linoleic (18:2n−6) acids accepted for teleosts^6^. Enzymatic activities shown in the diagram are predicted from heterologous expression in yeast (*Saccharomyces cerevisiae*) of fish fatty acyl desaturase 2 (Fads2) and Elongase of very long-chain fatty acid (Elovl) proteins. β-ox, partial β-oxidation

Long chain (≥C_20_) polyunsaturated fatty acids (LC-PUFA) including arachidonic acid (ARA, 20:4n−6), eicosapentaenoic acid (EPA, 20:5n−3) and docosahexaenoic acid (DHA, 22:6n−3) play numerous physiologically important roles essential to health in humans^1, 2^. Although humans have some ability to synthesise LC-PUFA from the C_18_ precursors linoleic acid (LOA, 18:2n−6) and α-linolenic acid (ALA, 18:3n−3), dietary supply of these LC-PUFA is still required to meet physiological demands^1^. Fish are the primary source of n−3 LC-PUFA for humans^3^ and this has prompted increasing interest in LC-PUFA metabolism in fish^4^, with biosynthesis being one of the most targeted pathways under investigation^5, 6^. The biosynthesis of C_20–22_ LC-PUFA in vertebrates including fish involves alternating steps of desaturation and elongation of the dietary essential C_18_ fatty acids (FA), LOA and ALA. Fatty acyl desaturases (Fads) catalyse the introduction of a double bond at a specific position of the acyl chain and have been named accordingly as ∆6, ∆5, ∆4 and ∆8 desaturases^7^. Elongation of very long-chain fatty acid (Elovl) proteins catalyse the condensation and rate-limiting reaction of the FA elongation pathway^8, 9^. Biosynthesis of ARA and EPA from the C_18_ precursors LOA and ALA, respectively, follows the same pathways and involves the same enzymes (Fig. 1). The pathways revealed from studies in vertebrates are the so-called “∆6 pathway” (∆6 desaturation − elongation − ∆5 desaturation) and the “∆8 pathway” (elongation − ∆8 desaturation − ∆5 desaturation) (Fig. 1)^6, 10–13^.

Since the studies of Sprecher and co-workers in rats^14–16^, it had been generally accepted that the biosynthesis of DHA in vertebrates was achieved by two consecutive elongations from EPA to produce tetracosapentaenoic acid (TPA, 24:5n−3), which then undergoes a ∆6 desaturation to tetracosahexaenoic acid (THA, 24:6n−3), the latter being β-oxidised to DHA in peroxisomes^17^. This pathway, known as the “Sprecher pathway”, was subsequently confirmed to be operative in rainbow trout *Oncorhynchus mykiss* ^18, 19^. The first question that arose after the demonstration of this pathway was whether the same or different ∆6 Fads catalysed the reactions with C_18_ and C_24_ substrates^15^. It was demonstrated that the same ∆6 Fads carried out the conversions of 18:3n−3 to 18:4n−3 and 24:5n−3 to 24:6n−3 in humans^20^ and rat^21, 22^. In fish species, it is still unclear whether the same Fads catalyses the two ∆6 desaturation reactions or if two ∆6 Fads (isoenzymes) are involved^12, 23, 24^. Studies using yeast as a heterologous expression system confirmed that the bifunctional ∆6∆5 Fads from zebrafish (*Danio rerio*) had ability to desaturate both C_18_ and C_24_ substrates at the ∆6 position^24^. However, the Nibe croaker (*Nibea mitsukurii*) ∆6 Fads catalysed the desaturation of C_18_ but not C_24_ substrates^25^. These findings suggested that the DHA biosynthetic capability varied among teleost fish and, interestingly, recent findings have demonstrated that, unlike other vertebrates, teleost fish have acquired alternative pathways for DHA biosynthesis during evolution^6^.

The “∆4 pathway”, first described in the marine protist *Thraustochytrium* sp.^26^, is a more direct pathway involving one single elongation of EPA to docosapentaenoic acid (DPA, 22:5n−3), which is subsequently desaturated at the ∆4 position to produce DHA. Although for many years Δ4 desaturases had not been found in any vertebrate species, a Fads2 with Δ4 desaturase activity was first discovered in rabbitfish (*Siganus canaliculatus*)^27^. Since then, Fads with ∆4 desaturases have been found in several teleost species such as Senegalese sole (*Solea senegalensis*)^28^, pike silverside (*Chirostoma estor*)^29^ and striped snakehead (*Channa striata*)^30^. Recently, human cells expressing the baboon *FADS2* had the ability for direct ∆4 desaturation of 22:5n−3 to 22:6n−3^31^. Thus, the existence of the ∆4 pathway among teleosts appeared to be more widespread than initially believed.

It is interesting to note that, unlike other vertebrates, current evidence suggests that all *fads*-like genes found in teleost fish are Fads2 orthologues^32^. Thus the functional diversity among fish Fads2 described above has been hypothesised to be dependent upon various factors including the phylogenetic position of species, in combination with environmental and ecological factors^6^. In the present study, we aimed to elucidate the pathways for DHA biosynthesis existing in species representing major lineages along the tree of life of teleost fish^33^. In particular, we have investigated the prevalence of the Sprecher pathway among teleost fish by determining the Δ6 activity towards C_24_ substrates (24:5n − 3 and 24:4n−6) of desaturases with different substrate specificities (Δ6, Δ5 and Δ4), and derived from fish species with different evolutionary and ecological backgrounds. Furthermore, we have taken advantage of the now known key amino acid (aa) residues determining Δ4 desaturase ability of Fads^34^ to identify teleost taxa, with publically available genomic or transcriptomic databases, in which their desaturase repertoire enables them to biosynthesise DHA through the more direct Δ4 pathway.

## Results

### Determination of Δ6 desaturase activity of fish Fads towards C_24_ PUFA

**Figure 2:**
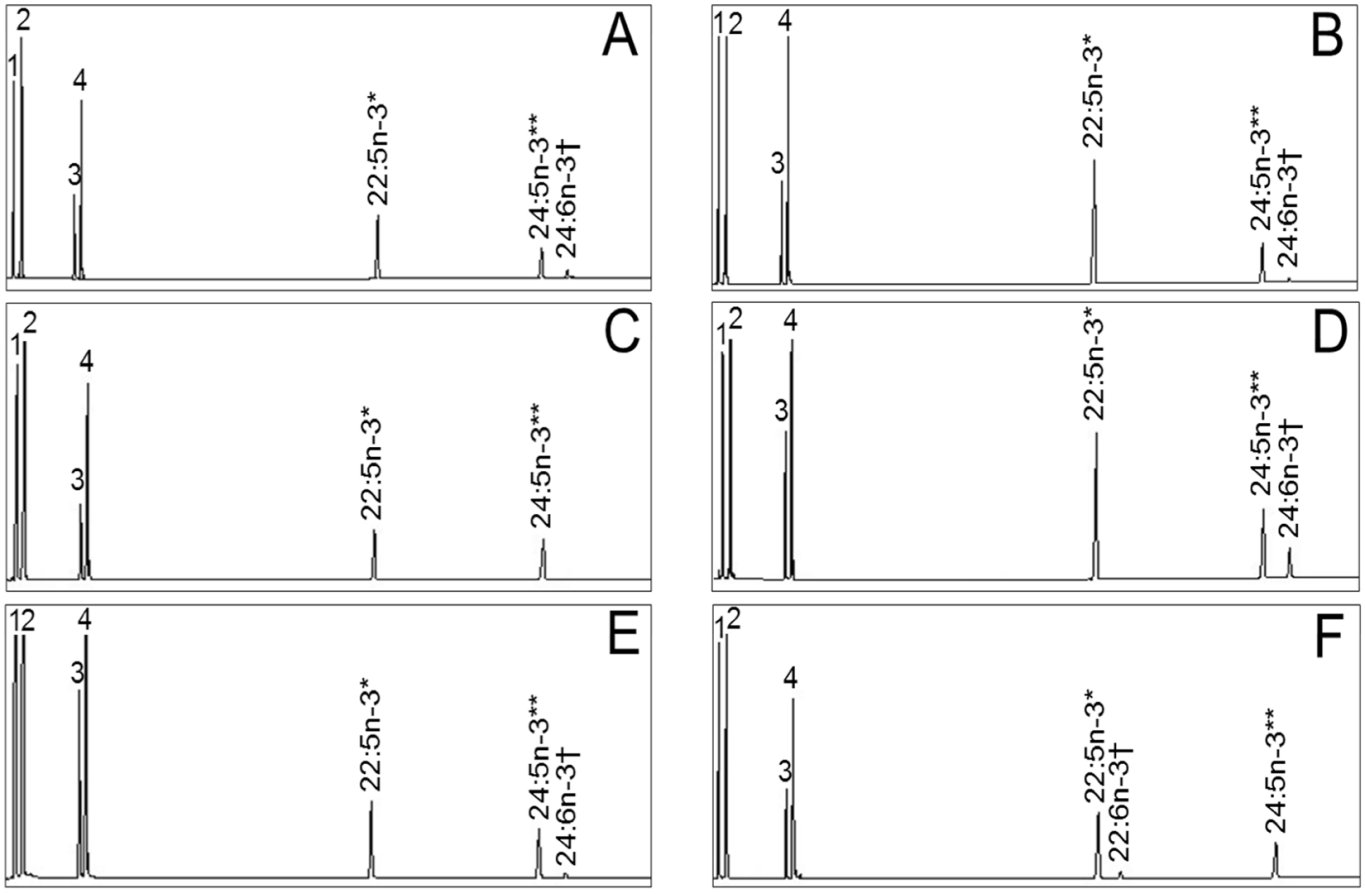
Characterisation of fish fatty acyl desaturases 2 (Fads2) ability to desaturate 24:5n−3. Fatty acid (FA) profiles of yeast (*Saccharomyces cerevisiae*) co-transformed with the *Danio rerio elovl2*, and the *Arapaima gigas* ∆6 *fads2* (**A**), *Sparus aurata* ∆6 *fads2* (**B**), *Nibea mitsukurii* ∆6 *fads2* (**C**), *Clarias gariepinus* ∆6∆5 *fads2* (**D**), *Salmo salar* ∆5 *fads2* (**E**) and *Chirostoma estor* ∆4 *fads2* (**F**) and grown in the presence of an exogenously added FA substrates (indicated as “*” in all panels). Peaks 1–4 represent the *S. cerevisiae* endogenous FA, namely 16:0 (1), 16:1 isomers (2), 18:0 (3) and 18:1n-9 (4). Elongation (**) and desaturation (†) products from exogenously added or endogenously produced FA are indicated accordingly.

The capabilities of fish Fads to desaturate C_24_ PUFA (24:4n−6 and 24:5n−3) at Δ6 position were determined by co-transforming yeast with *D. rerio elovl2* and the individual fish *fads* to be assayed. Control yeast co-transformed with empty p415TEF and pYES2 vectors did not show any activity towards any of the PUFA substrates assayed (data not shown) and the yeast showed typical FA profiles consisting primarily of 16:0, 16:1 isomers, 18:0 and 18:1n-9 (Fig. 2). Independent of the desaturase cloned into the inducible expression vector pYES2, all the co-transformant yeast were able to elongate the exogenously added 22:4n−6 and 22:5n−3 to 24:4n−6 and 24:5n−3, respectively, confirming the activity of the *D. rerio* Elovl2 cloned into the constitutive expression vector p415TEF. Importantly, the incubation of all the co-transformant yeast in the presence of the corresponding FA substrate as controls (i.e. 18:3n−3 for Δ6 and Δ6Δ5 desaturases, 20:4n−3 for Δ5 desaturases, and 22:5n−3 for Δ4 desaturases) confirmed that the desaturases were functional, with activities as previously reported (Table 1).

**Table 1:** Capability of fish Fads2 for Δ6 desaturation of C_24_ substrates 24:4n−6 and 24:5n−3 using a yeast *Saccharomyces cerevisiae* heterologous system as described in Materials and Methods. Fatty acid (FA) conversions were calculated as the percentage of 24:4n−6 and 24:5n−3 desaturated to 24:5n−6 and 24:6n−3, respectively, as [product area/(product area + substrate area)] × 100. Conversions towards the control FA substrate (18:3n−3 as controls for Δ6 and Δ6Δ5 desaturases, 20:4n−3 for Δ5 desaturases and 22:5n−3 for Δ4 desaturases) are also indicated. In order to normalise the % conversions obtained throughout the Fads2 dataset, ratios between the activities on 24:5n−3 and those on the control FA (“Δ_24:5n−3_/Δ_control_”) are also presented.

Desaturase^a^	% Conversion			
	24:4n−6 → 24:5n−6	24:5n−3 → 24:6n−3	Control → Product	Δ_24:5n−3_/Δ_control_
ScyΔ6Fads2	29.3	34.3	41.9	0.82
AgΔ6Fads2	25.4	19.0	15.3	1.24
AjΔ6Fads2	14.0	15.8	17.8	0.89
DrΔ6Δ5Fads2	10.4	15.8	11.9	1.33
CgΔ6Δ5Fads2	29.9	28.1	31.5	0.89
SsΔ6Fads2	18.5	26.0	23.9	1.09
SsΔ5Fads2	1.4	6.4	3.4	1.88
OmΔ6Fads2	7.5	19.7	20.4	0.97
CeΔ6Δ5Fads2	4.2	9.0	22.9	0.39
CeΔ4Fads2	ND	ND	9.9	0.00
ScΔ6Δ5Fads2	6.0	7.4	36.4	0.20
ScΔ4Fads2	ND	ND	6.9	0.00
SaΔ6Fads2	4.8	6.5	15.0	0.43
NmΔ6Fads2	ND	ND	10.5	0.00
OnΔ4Fads2	ND	ND	4.5	0.00

ND, Not detected

^a^Scy, *Scyliorhinus canicula;* Ag, *Arapaima gigas*; Aj, *Anguilla japonica*; Dr, *Danio rerio*; Cg, *Clarias gariepinus*; Ss, *Salmo salar*; Om, *Oncorhychus mykiss*; Ce, *Chirostoma estor*; Sc, *Siganus canaliculatus*; Sa, *Sparus aurata*; Nm, *Nibea mitsukurii*; On, *Oreochromis niloticus*.

The ability for Δ6 desaturation of C_24_ PUFA such as 24:4n−6 and 24:5n−3 varied among fish Fads (Fig. 2; Table 1). Interestingly, none of the three Δ4 Fads2 assayed (*C. estor*, *S. canaliculatus* and *Oreochromis niloticus*) showed any ability to desaturate either 24:4n−6 or 24:5n−3 (Table 1). However, most of the fish Fads2 with Δ6 and/or Δ5 specificities were capable of desaturating both 24:4n−6 and 24:5n−3 to their corresponding Δ6 desaturated products, namely 24:5n−6 and 24:6n−3, respectively (Fig. 2; Table 1). Due to the intrinsic variability of desaturation activities in the yeast system, we normalised the Δ6 desaturase activities measured on C_24_ substrates with those obtained on the corresponding control FA substrate. For that purpose, we calculated the ratio “Δ_24:5n−3_/Δ_control_” (Table 1) that allowed comparisons among the fish Fads investigated herein. Generally, desaturases from species within relatively ancient fish lineages including *Scyliorhinus canicula*, *Arapaima gigas*, *Anguilla japonica*, *Clarias gariepinus*, *Salmo salar* and *O. mykiss* showed high capacity for Δ6 desaturation towards 24:5n−3, with Δ_24:5n−3_/Δ_control_ ratios ≥ 0.82 (Table 1). On the other hand, more modern species such as *S. canaliculatus*, *Sparus aurata* and *N. mitsukurii* had Fads2 with Δ_24:5n−3_/Δ_control_ ratios ≤ 0.43 (Table 1). It is interesting to note that the *S. salar* Δ5 (SsΔ5Fads2) showed the ability to desaturate 24:5n−3 to 24:6n−3 (Fig. 2E; Table 1), denoting Δ6 desaturase activity. In order to confirm these results, we incubated the SsΔ5Fads2 co-transformant yeast in the presence of 18:3n−3 and confirmed the presence of Δ6 desaturated product 18:4n−3 (3.5% conversion). Among all the non-Δ4 Fads2, the *N. mitsukurii* NmΔ6Fads2 was the only tested desaturase with no activity on either 24:4n−6 nor 24:5n−3 (Fig. 2C).

### Putative Δ4 desaturase collection and phylogenetics

**Figure 3:**
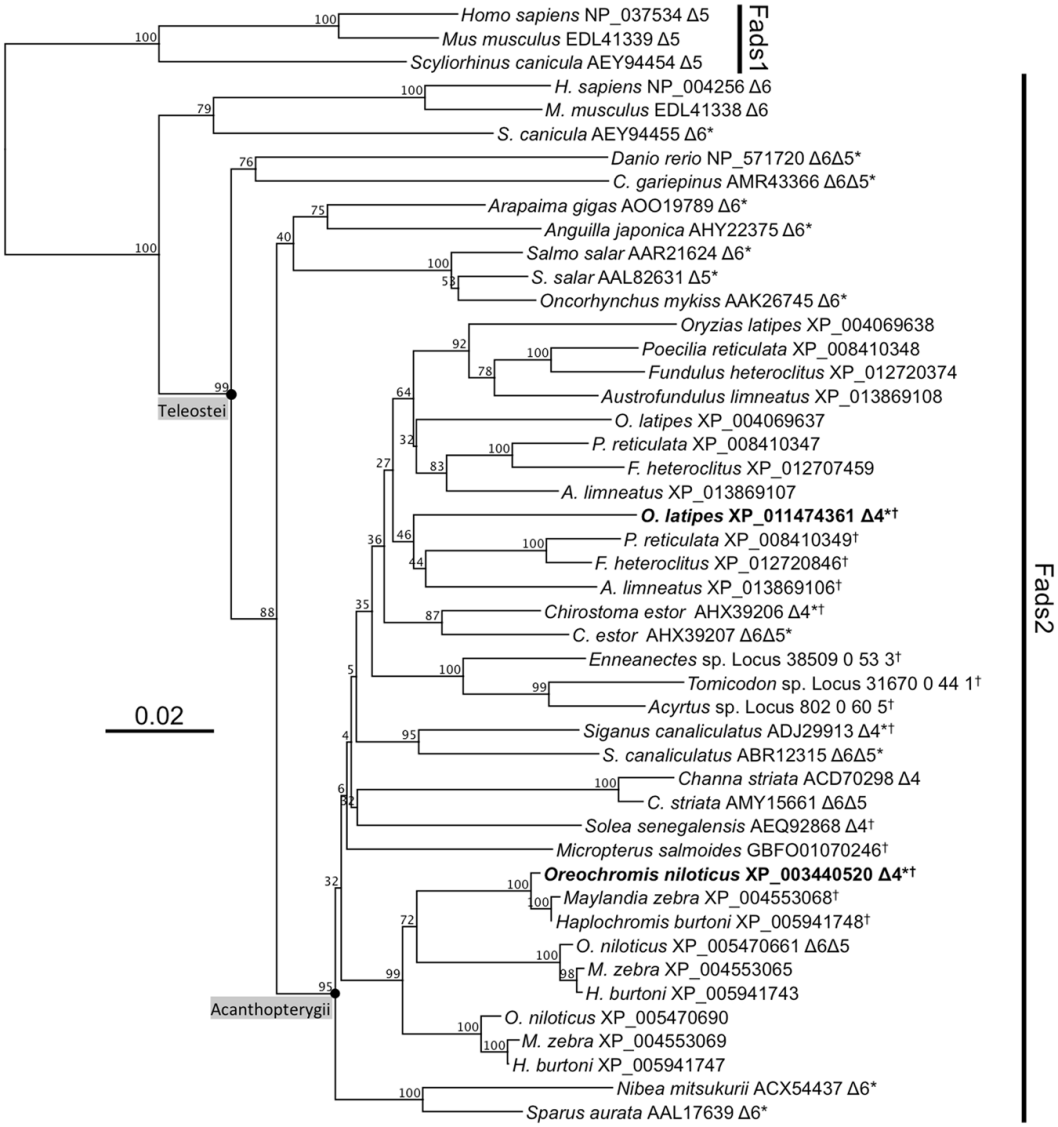
Phylogenetic tree comparing the amino acid sequences of teleost Fads2 with non-teleost vertebrate Fads-like from the cartilaginous fish and mammals (human and mouse). The numbers represent the frequencies (%) with which the tree topology presented was replicated after 1,000 iterations. The functionally characterised Fads were shown with their corresponding regioselectivity (Δ6, Δ5, Δ6Δ5 and Δ4). Asterisks (“*”) indicate Fads2 that have been subjected to further functional analyses in the present study, including the newly cloned Δ4 Fads2 from medaka (*Oryzias latipes*) and Nile tilapia (*Oreochromis niloticus*) highlighted in bold. Crosses (“†”) indicate Fads2 that possess the YXXN amino acid residues determining Δ4 desaturase activity^32^. Branches including Teleostei and Acanthopterygii Fads2 sequences are indicated.

The phylogenetic tree comparing the deduced aa sequence of the fish Fads with those of human and rat is shown in Fig. 3. All Fads1 clustered together and were separate from all Fads2 in the tree. All teleost Fads2 studied in the present study strongly clustered within the teleost group (99% bootstraps), with desaturases from early divergent teleost species (e.g. *A. gigas*, *A. japonica*, *C. gariepinus*, *S. salar* and *O. mykiss*) clustering separately from desaturases from species belonging to more recent lineages (95% bootstraps) (Fig. 3). Among the latter, one can find all the sequences with YXXN residues determining Δ4 activity^34^ including the previously studied Δ4 desaturases from *S. canaliculatus*, *S. senegalensis* and *C. estor* and the herein characterised Fads2 from medaka (*Oryzias latipes*) and Nile tilapia (*O. niloticus*). Clearly, all Fads2-like proteins from Nile tilapia and other cichlids formed a monophyletic clade (99% bootstraps), itself comprising a subgroup with Fads2 sequences possessing the abovementioned distinctive YXXN motif for Δ4 desaturases and another group that includes the Δ6Δ5 Fads2 from Nile tilapia (Fig. 3).

### Functional characterisation of Oryzias latipes and Oreochromis niloticus Δ4 desaturase

**Figure 4:**
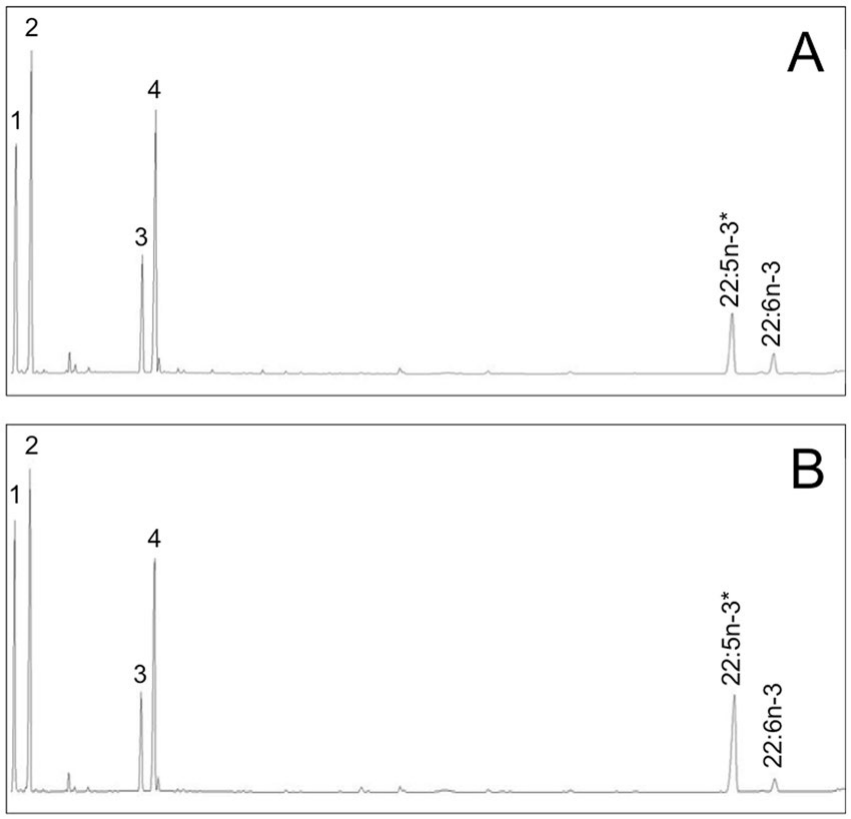
Δ4 desaturase activity towards 22:5n−3 of the newly cloned *fads2* from *Oryzias latipes* (**A**) and *Oreochromis niloticus* (**B**). Peaks 1–4 represent *Saccharomyces cerevisiae* endogenous FA, namely 16:0 (1), 16:1 isomers (2), 18:0 (3) and 18:1n-9 (4). Peaks derived from exogenously added substrates (*) and the desaturation product 22:6n−3 (DHA) are indicated accordingly.

In order to confirm the function of the putative Δ4 desaturases retrieved by *in silico* searches, we selected those from medaka *O. latipes* (XM_011476059) and Nile tilapia *O. niloticus* (XM_003440472) and characterised their function in yeast. Both desaturases were able to desaturate 22:5n−3 and 22:4n−6 to 22:6n−3 (DHA) and 22:5n−6, respectively (Fig. 4; Table 2), demonstrating that they were both Δ4 desaturases as predicted. Interestingly, both enzymes showed some activity as Δ5 desaturases, since they were able to convert 20:4n−3 and 20:3n−6 into 20:5n−3 (EPA) and 20:4n−6 (ARA), respectively (Table 2). Generally, the Nile tilapia Δ4 Fads2 showed higher conversion activities compared to that of medaka and both proteins were more efficient towards n−3 substrates compared to n−6 substrates.

**Table 2:** Substrate conversions of *Saccharomyces cerevisiae* transformed with *Oryzias latipes* and *Oreochromis niloticus fads2* coding region and grown in the presence of one exogenously added fatty acid (FA) substrate (18:3n−3, 18:2n−6, 20:4n−3, 20:3n−6, 22:5n−3 or 22:4n−6). Conversions were calculated according to the formula [product area/(product area + substrate area)] × 100.

Species	FA substrate	FA product	Conversion (%)	Activity
*O. latipes*	18:3n−3	18:4n−3	ND	Δ6
	18:2n−6	18:3n−6	ND	Δ6
	20:4n−3	20:5n−3	11.8	Δ5
	20:3n−6	20:4n−6	2.3	Δ5
	22:5n−3	22:6n−3	24.1	Δ4
	22:4n−6	22:5n−6	13.7	Δ4
*O. niloticus*	18:3n−3	18:4n−3	ND	Δ6
	18:2n−6	18:3n−6	ND	Δ6
	20:4n−3	20:5n−3	1.6	Δ5
	20:3n−6	20:4n−6	0.3	Δ5
	22:5n−3	22:6n−3	10.8	Δ4
	22:4n−6	22:5n−6	8.1	Δ4

ND, Not detected

## Discussion

It has been largely accepted that DHA biosynthesis in vertebrates proceeds through the Sprecher pathway^6, 9^. While most earlier investigations focussed on mammals, studies in *O. mykiss* confirmed that the Sprecher pathway also operated in fish^18, 19^. It was subsequently demonstrated that the same ∆6 Fads-like enzyme that acts on C_18_ PUFA precursors at the initiation of the LC-PUFA biosynthesis (Fig. 1) was also responsible for the desaturation of 24:5n−3 required in the Sprecher pathway^20, 22^. Despite the plethora of studies reporting on the functions of fish Fads^6^, the capability of fish Fads to operate towards 24:5n−3, and therefore to contribute to DHA biosynthesis through the Sprecher pathway, had not been fully established. For that purpose, we herein conducted a retrospective study investigating the ability to operate as ∆6 desaturases towards 24:5n−3 and 24:4n−6 of a range of previously characterised Fads2 from fish belonging to lineages distributed along the phylogenetic tree of teleosts^33^.

Using a newly developed method involving yeast, we were able to establish that, with the exception of the Nibe croaker Fads2, all teleost non-∆4 desaturases tested in this study had the ability to efficiently convert 24:5n−3 and 24:4n−6 into 24:6n−3 and 24:5n−6, respectively, confirming their ability for ∆6 desaturation of C_24_ PUFA substrates. Such ability was observed in Fads2 from species spread across the evolutionary history of teleosts from basal (e.g. *A. gigas* and *A. japonica*) and recent (e.g. *S. canaliculatus* and *S. aurata*) lineages, and with different regioselectivities including ∆6 desaturases (e.g. *O. mykiss* and *S. aurata*) and bifunctional ∆6∆5 desaturases (e.g. *C. estor* and *S. canaliculatus*). Since all these Fads2 also showed ∆6 desaturase activity towards C_18_ PUFA (18:3n−3 and 18:2n−6), the present results confirmed that the same Fads2 can function as ∆6 desaturases at both steps of the LC-PUFA biosynthetic pathway as described above for mammals^20, 22^. This is in agreement with studies on zebrafish ∆6∆5 Fads2, which showed the ability to operate as ∆6 desaturase on C_18_^35^ and C_24_ PUFA substrates, using for the latter a yeast supplemented with 24:5n−3^24^. Interestingly, we could also confirm that the previously characterised ∆5 Fads2 from Atlantic salmon *S. salar* ^36^, also showed ∆6 activity on C_24_ PUFA (24:4n−6 and 24:5n−3) and 18:3n−3, suggesting that this enzyme is indeed a bifunctional ∆6∆5 desaturase. Bifunctionality appears a relatively widespread feature among fish Fads2 as a consequence of sub- (acquisition of additional substrate specificities) and neo-functionalisation (substitution and/or acquisition of new substrate specificities) events that have occurred in teleost Fads2^6, 29^. More specifically, bifunctional ∆6∆5 Fads2 have been found in *D. rerio* ^35^, *S. canaliculatus* ^27^, *O. niloticus* ^37^, *C. estor* ^29^, *C. gariepinus* ^38^ and *C. striata* ^39^. Moreover, all the ∆4 Fads2 found so far in fish also exhibited some ∆5 desaturase activity^27–30^, although none of them had ∆6 activity, which is consistent with the lack of ∆6 desaturase activity towards C_24_ PUFA substrates observed in all the ∆4 Fads2 assayed in the present study. Interestingly, our results show that the two pathways of DHA biosynthesis, namely the Sprecher and ∆4 pathways, co-exist within some species such as *S. canaliculatus* and *C. estor* since, in addition to the role of their ∆6∆5 Fads2 in the Sprecher pathway uncovered in the present study, the existence of ∆4 desaturases in their genomes potentially enables them to further operate via the ∆4 pathway^27, 29^.

The Δ4 pathway was first reported in the rabbitfish *S. canaliculatus* ^27^, with further Δ4 desaturases subsequently described in *S. senegalensis*, *C. estor* and *C. striata* ^28–30^. In the present study, we have expanded the number of fish lineages and species in which putative Δ4 desaturases exist. In particular, putative Δ4 desaturases were identified in 11 species belonging to Cichliformes (*O. niloticus*, *Maylandia zebra* and *Haplochromis burtoni*), Beloniformes (*O. latipes*), Blenniiformes (*Tomicodon* sp., *Acyrtus* sp. and *Enneanectes* sp.), Cyprinodontiformes (*Poecilia reticulata*, *Fundulus heteroclitus* and *Austrofundulus limnaeus*) and Centrarchiformes (*Micropterus salmoides*). It is very likely that the number of species with Δ4 Fads2 will expand when further genomic and/or transcriptomic data become available. This is particularly true for species within groups such as Cichliformes and Beloniformes, in which we found putative Δ4 Fads2 in all species studied in each group. While the actual functional activity of the retrieved desaturases should be assessed in each individual species, the characterisation of two of the newly identified putative Δ4 desaturases, namely the *O. latipes* and *O. niloticus* Fads2, as functional Δ4 enzymes supported the effectiveness of our *in silico* search strategy using a conserved aa region containing the YXXN motif responsible for Δ4 activity to identify functional Δ4 desaturases^34^. Overall, these results clearly showed that the presence of Δ4 Fads2 among teleosts was far more common than initially believed when the first vertebrate Δ4 desaturase was discovered in *S. canaliculatus* ^27^. However, the presence of Δ4 Fads2 appears to be restricted to teleost species within groups regarded herein as “recent lineages”, indicating that the acquisition of the Δ4 pathway occurred later during the evolution of teleosts^6, 29^.

In more basal teleost lineages, namely Osteoglossiformes (e.g. *A. gigas*), Anguilliformes (e.g. *A. japonica*), Cypriniformes (e.g. *D. rerio*), Siluriformes (e.g. *C. gariepinus*) and Salmoniformes (e.g. *S. salar* and *O. mykiss*), the Sprecher pathway appears to be the only possible route available for DHA biosynthesis. This is supported by, not only the apparent absence of Δ4 Fads2 in their genomes, but also the relatively higher capacity for Δ6 desaturase towards 24:5n−3 of their Fads2, as denoted by normalising the Δ6 conversions of 24:5n−3 (Δ_24:5n−3_) with that towards a control substrate (Δ_control_). Thus, Fads2 from early divergent teleosts, along with the cartilaginous fish *S. canicula*, had relatively high capacity for ∆6 desaturation towards 24:5n−3, with Δ_24:5n−3_/Δ_control_ ≥ 0.82. In contrast, Fads2 from other species (*S. aurata*, *C. estor* and *S. canaliculatus*) had lower Δ_24:5n−3_/Δ_control_ ≤ 0.43, indicating lower activity of the Sprecher pathway. While exceptions to this pattern are likely to exist given the functional diversity among teleost Fads2^6, 29^, the apparent lower contribution of the Sprecher pathway to DHA biosynthesis in late-diverging teleosts coincided with the occurrence of Δ4 Fads2 enabling certain species an alternative route for DHA biosynthesis. The limited activity of the Sprecher pathway among these teleost species might be not only restricted to their lower desaturation capability on 24:5n−3 stated above, but also to the absence of key elongase enzymes such as Elovl2, responsible for the production of the Δ6 desaturase substrate 24:5n−3^40–42^. Although Elovl4 can partly compensate such an absence in certain tissues^42–45^, loss of Elovl2 in the genomes of Acanthopterygii, a group that includes all the late-diverging species considered in this study^46^, can notably compromise the efficient production of 24:5n−3 as precursor for DHA biosynthesis via the Sprecher pathway. Lack of key enzymatic capabilities in LC-PUFA biosynthetic pathways has been speculated to be a consequence of species having readily available essential LC-PUFA in their diets^24, 41^. This is the case of marine teleosts, particularly higher trophic species, in which no selection pressure to retain complete and active LC-PUFA biosynthetic pathways has been exerted. For example, extreme cases of marine teleosts with loss of enzymatic activities include the pufferfish (e.g. *Tetraodon nigroviridis* and *Takifugu rubripes*), which lack Fads2 in their genomes^46^. In the present study, we observed that the marine carnivore Nibe croaker *N. mitsukurii* possess a Fads2 that was the only non-Δ4 Fads2 studied that showed no detectable activity towards 24:5n−3. These results were consistent with the inability of *N. mitsukurii* Fads2 to desaturate 24:5n−3 to 24:6n−3 in yeast^25^ and the accumulation of 24:5n−3, but not DHA, in transgenic *N. mitsukurii* carrying an *elovl2* ^47^.

## Conclusions

The present study demonstrated that, with the notable exception of Δ4 desaturases, fish Fads2 have the ability to operate as Δ6 desaturases towards C_24_ PUFA enabling them to synthesise DHA through the Sprecher pathway. However, the so-called “Δ4 pathway” represents an alternative route in some species. Through *in silico* searches, the present study revealed that the presence of Δ4 Fads was more common than initially believed, and reported three new orders and 11 species in which putative Δ4 desaturases were identified. Importantly, two putative Δ4 Fads2 desaturases retrieved from medaka and Nile tilapia *in silico* were confirmed as functional Δ4 enzymes. Interestingly, functional characterisation of the *S. salar* Fads2 previously characterised as a Δ5 desaturase confirmed this enzyme has also Δ6 desaturase activity and should be therefore regarded as a bifunctional Δ6Δ5 desaturase. Overall our results demonstrate that two alternative routes for DHA biosynthesis can exist in teleost fish. Whereas the Sprecher pathway appeared to be widely spread across the entire clade, a more scattered distribution was observed for the Δ4 pathway.

## Methods

### Fish lineages

A comprehensive set of Fads2-like sequences was collected by screening genomic and transcriptomic databases from fish species representing a sample group of lineages such as the basal gnathostome *S. canicula*; early diverging post-3R teleosts Osteoglossiformes (*A. gigas*) and Anguilliformes (*A. japonica*); and various other teleostei such as Cypriniformes (*D. rerio*), Siluriformes (*C. gariepinus*) and Salmoniformes (*S. salar* and *O. mykiss*), to relatively modern groups like Anabantiformes (*C. striata*), Atheriniformes (*C. estor*), Cichliformes (*O. niloticus*, *M. zebra* and *H. burtoni*), Blenniiformes (*Tomicodon* sp., *Acyrtus* sp. and *Enneanectes* sp.), Beloniformes (*O. latipes*), Cyprinodontiformes (*P. reticulata*, *F. heteroclitus* and *A. limnaeus*), Pleuronectiformes (*S. senegalensis*), Spariformes (*S. aurata*), Centrarchiformes (*M. salmoides*) and Eupercaria (*S. canaliculatus* and *N. mitsukurii*). The desaturase sequences from fish species listed above were used for phylogenetic analysis and selected sequences were subjected to functional characterisation as described.

### Determination of Δ6 desaturase activity of fish Fads2 towards C_24_ PUFA in co-transformant Saccharomyces cerevisiae

We first investigated the ability for Δ6 desaturase activity towards C_24_ PUFA substrates, i.e. 24:4n−6 and 24:5n−3, the latter being an intermediate in the Sprecher pathway for DHA biosynthesis. Such activities were tested in a total of 15 Fads sequences belonging to 12 species of fish (Table 3), through a newly developed yeast-based assayed as follows. Yeast competent cells Inv*Sc*1 (Invitrogen) were co-transformed with two different plasmid constructs prepared as described below. First, the *D. rerio elovl2* open reading frame (ORF)^48^ was ligated into the yeast expression vector p415TEF (a centromeric plasmid with a *LEU2* selectable marker) to produce the construct p415TEF-*elovl2*, in which the expression of the *D. rerio elovl2* was controlled under the yeast *TEF1* promoter (constitutive expression). Second, the ORF of the corresponding fish Fads (Table 3) was cloned into the episomal yeast vector pYES2, in which the Fads expression was under the control of the *GAL1* promoter (inducible expression). Selection of transformant yeast containing both constructs was performed by growing the co-transformed yeast on *S. cerevisiae* minimal medium minus uracil minus leucine (SCMM^−ura−leu^) plates. One single colony was grown in SCMM^−ura−leu^ broth for 24 h at 30 °C, and subsequently subcultured in individual Erlenmeyer flasks at 0.1 OD_600_ (t_0_) and supplemented with either 0.75 mM Na salts of 22:4n−6 (docosatetraenoic acid, DTA) or 22:5n−3 (DPA) (0.75 mM). Co-transformed yeast were then grown for 24 h (t_0_ + 24 h) allowing the *D. rerio* Elovl2 to convert the exogenously added C_22_ substrates (DTA or DPA) into their corresponding C_24_ elongation products 24:4n−6 and 24:5n−3, respectively. In order to test the ability of the fish desaturases to introduce Δ6 double bonds into the newly synthesised 24:4n−6 and 24:5n−3 in yeast, the *fads* expression was then induced (t_0_ + 24 h) by addition of 2% galactose, after which the recombinant yeast were further grown for 48 h (t_0_ + 72 h) before collection. As positive controls, a subculture aliquot of the same colony used for the above described assay was supplemented with an n−3 PUFA substrate for which the corresponding assayed Fads had previously shown activity (Table 3) and galactose (2%) at t_0_. More specifically, co-transformant yeasts were grown in the presence of 18:3n−3 as controls for Δ6 (e.g. AgΔ6Fads2) or Δ6Δ5 (e.g. DrΔ6Δ5Fads2) desaturases, 20:4n−3 for Δ5 desaturases (e.g. SsΔ5Fads2) and 22:5n−3 for Δ4 desaturases (e.g. CeΔ4Fads2). The yeast co-transformed with empty p415TEF and pYES2 vectors were also prepared as negative controls.

**Table 3:** Fish fatty acyl desaturases (Fads) investigated for the ability to desaturate tetracosapentaenoic acid (24:5n−3) to tetracosahexaenoic acid (24:6n−3). Their known desaturation activities and the studies in which they were published are indicated accordingly.

Species	Desaturase name^a^	Reported activity^b^	GenBank Accession no.	Reference
*Scyliorhinus canicula*	ScyΔ6Fads2	Δ6	JN657544	32
*Arapaima gigas*	AgΔ6Fads2	Δ6	AOO1978	51
*Anguilla japonica*	AjΔ6Fads2	Δ6	AHY22375	53
*Danio rerio*	DrΔ6Δ5Fads2	Δ6, Δ5	AAG25710	35
*Clarias gariepinus*	CgΔ6Δ5Fads2	Δ6, Δ5	AMR43366	38
*Salmo salar*	SsΔ6Fads2	Δ6^c^	AAR21624	54
*S. salar*	SsΔ5Fads2	Δ5	AAL82631	36
*Oncorhynchus mykiss*	OmΔ6Fads2	Δ6	AAK26745	54
*Chirostoma estor*	CeΔ6Δ5Fads2	Δ6, Δ5	AHX39207	29
*C. estor*	CeΔ4Fads2	Δ4	AHX39206	29
*Siganus canaliculatus*	ScΔ6Δ5Fads2	Δ6, Δ5	ABR12315	27
*S. canaliculatus*	ScΔ4Fads2	Δ4	ADJ29913	27
*Sparus aurata*	SaΔ6Fads2	Δ6	AAL17639	55
*Nibea mitsukurii*	NmΔ6Fads2	Δ6	AJD80650	25
*Oreochromis niloticus*	OnΔ4Fads2	Δ4^d^	XP_003440520	Present study

^a^Scy, Scyliorhinus canicula; Ag, Arapaima gigas; Aj, Anguilla japonica; Dr, Danio rerio; Cg, Clarias gariepinus; Ss, Salmo salar; Om, Oncorhychus mykiss; Ce, Chirostoma estor; Sc, Siganus canaliculatus; Sa, Sparus aurata; Nm, Nibea mitsukurii; On, Oreochromis niloticus. ^b^Δ8 desaturase activities of some of these desaturases and reported in the corresponding publication are not indicated in the interests of clarity. ^c^Refers to “Fads2_a” as termed by Monroig *et al*.^56^. ^d^Functional characterisation of OnΔ4Fads2 was carried out in the present study.

### *In silico* retrieval of putative Δ4 desaturases

For retrieval of putative Δ4 desaturase sequences from databases, an alignment of the four functionally characterised Δ4 desaturases from rabbitfish (ADJ29913), Senegalese sole (AEQ92868), pike silverside (AHX39206) and striped snakehead (ACD70298) was performed using the Clustal Omega Multiple Sequence Alignment tool (http://www.ebi.ac.uk/Tools/msa/clustalo/). The conserved aa sequence PPLLIPVFYNFNIMXTMISR, which included the four key aa residues (underlined) accounting for Δ4 regioselectivity^34^, was used as a query for blast searches. The majority of the putative Δ4 desaturase sequences were obtained from the NCBI Non-redundant protein sequences (nr) database using the blastp algorithm. We further explored the Expressed Sequence Tags (EST) and Transcriptome Shotgun Assembly (TSA) databases using the tblastn algorithm. In addition, the Fish-T1K website (http://www.fisht1k.org) was also used for the tblastn search. Among the retrieved sequences, we selected only those that contained “Y” and “N” in positions 1 and +4, respectively, within the four aa domain YXXN, as these have been reported previously to be crucial for Δ4 function^34^.

### Phylogenetic analysis of Fads desaturases

A phylogenetic tree was built to compare the deduced aa sequences of the fish Fads considered in the present study. The neighbour-joining method^49^, with the CLC Main Workbench 7 (CLC bio, Aarhus, Denmark), was used to construct the phylogenetic tree, with confidence in the resulting tree branch topology measured by bootstrapping through 1,000 iterations. The alignment of Fads aa sequences used for constructing the phylogenetic tree was performed with MAFFT using the L-INS-i method^50^. Non-teleost fish sequences from *S. canicula* and mammalian (human and mouse) Fads2 sequences were also included in the analysis.

### *O. latipes* and *O. niloticus* putative Δ4 desaturases: Molecular cloning and functional characterisation by heterologous expression in Saccharomyces cerevisiae

In order to confirm that the *in silico* retrieved Fads sequences encoded Δ4 desaturases, we performed the functional analysis of those identified in *O. latipes* (XM_011476059) and *O. niloticus* (XM_003440472). The ORF of the putative Δ4 desaturase sequences were amplified using as template cDNA prepared from a mixture of liver, brain and intestine RNA samples and with primers containing *Bam*HI and *Xho*I restriction sites (underlined). The primers for *O. latipes* putative Δ4 Fads2 were CCCGGATCCAAGATGGGAGGTGGAGGTCA (forward) and CCGCTCGAGTCATTTATGAAGATATGCATCAAGC (reverse), whereas the primers CCCGGATCCAGGATGGGACGTGGAAGC (forward) and CCGCTCGAGTCATTTATGGAGGTAAGCGT (reverse) were used to amplify *O. niloticus* putative Δ4 Fads2. For both genes, PCR were performed using the Phusion HF polymerase (Thermo Fisher Scientific, Waltham, MA, USA) with an initial denaturing step at 98 °C for 30 s, followed by 35 cycles of denaturation at 98 °C for 10 s, annealing at 55 °C for 30 s, extension at 72 °C for 1 min followed by a final extension at 72 °C for 10 min. The DNA fragments of the *O. latipes* and *O. niloticus fads2* obtained were purified, digested with the restriction enzymes, and ligated into similarly digested pYES2 yeast expression vector (Invitrogen) producing the constructs pYES2-*Olfads2* and pYES2-*Onfads2*, respectively. Yeast competent cells InvSc1 (Invitrogen) were transformed with the plasmid constructs using the *S.c*. EasyComp^TM^ Transformation Kit (Invitrogen). Selection of yeast containing the pYES2 constructs was performed on *S. cerevisiae* minimal medium minus uracil (SCMM^−ura^) plates. One single yeast colony of each transformation (pYES2-*Olfads2* or pYES2-*Onfads2*) was grown in SCMM^−ura^ broth for 2 days at 30 °C, and subsequently subcultured in individual Erlenmeyer flasks until optical density measured at a wavelength of 600 nm (OD_600_) reached 1, after which galactose (2%, w/v) and a PUFA substrate were added as sodium (Na) salts. The exogenous supplemented PUFA included Δ6 (18:3n−3 and 18:2n−6), Δ5 (20:4n−3 and 20:3n−6), and Δ4 (22:5n−3 and 22:4n−6) desaturase substrates, at final concentrations of 0.5 mM (C_18_), 0.75 mM (C_20_) and 1.0 mM (C_22_) to compensate for differential uptake related to fatty acyl chain^51^. After 2 days, transgenic yeast expressing either the *O. latipes* or *O. niloticus fads2* were harvested and processed for fatty acid analysis as below. All FA substrates (>98–99% pure) used for the functional characterisation assays, except for stearidonic acid (18:4n−3) and eicosatetraenoic acid (20:4n−3), were obtained from Nu-Chek Prep, Inc. (Elysian, MN, USA). Stearidonic acid (>99% pure) and yeast culture reagents including galactose, nitrogen base, raffinose, tergitol NP-40 and uracil dropout medium were obtained from Sigma-Aldrich (UK). Eicosatetraenoic acid was purchased from Cayman Chemical Co. (Ann Arbor, USA).

### Fatty acid analysis of yeast

Total lipids extracted from yeast samples^52^ were used to prepare fatty acid methyl esters (FAME). FAME extraction, purification and analysis were performed as described by Li *et al*.^27^. For functional characterisation of the desaturases from *O. latipes* and *O. niloticus*, substrate FA conversions were calculated as the proportion of exogenously added FA substrate desaturated as [product area/(product area + substrate area)] × 100^51^. Substrate FA conversions for the Δ6 desaturase activity towards C_24_ substrates were calculated using the same formula as above considering the areas of 24:5n−3 and 24:4n−6 produced endogenously by the *D. rerio* Elovl2 as substrates for calculations. When necessary, GC-MS was used to confirm the identity of the products^27^.
